# Structural Details of BH3 Motifs and BH3-Mediated Interactions: an Updated Perspective

**DOI:** 10.3389/fmolb.2022.864874

**Published:** 2022-05-24

**Authors:** Valentina Sora, Elena Papaleo

**Affiliations:** ^1^ Cancer Structural Biology, Danish Cancer Society Research Center, Copenhagen, Denmark; ^2^ Cancer Systems Biology, Section for Bioinformatics, Department of Health and Technology, Technical University of Denmark, Kongens Lyngby, Denmark

**Keywords:** short linear motifs, protein-protein interactions, disordered proteins, BCL2, apoptosis

## Abstract

Apoptosis is a mechanism of programmed cell death crucial in organism development, maintenance of tissue homeostasis, and several pathogenic processes. The B cell lymphoma 2 (BCL2) protein family lies at the core of the apoptotic process, and the delicate balance between its pro- and anti-apoptotic members ultimately decides the cell fate. BCL2 proteins can bind with each other and several other biological partners through the BCL2 homology domain 3 (BH3), which has been also classified as a possible Short Linear Motif and whose distinctive features remain elusive even after decades of studies. Here, we aim to provide an updated overview of the structural features characterizing BH3s and BH3-mediated interactions (with a focus on human proteins), elaborating on the plasticity of BCL2 proteins and the motif properties. We also discussed the implication of these findings for the discovery of interactors of the BH3-binding groove of BCL2 proteins and the design of mimetics for therapeutic purposes.

## Introduction

Under physiological conditions, dysfunctional or no longer necessary cells are cleared *via* pathways of regulated cell death, among which apoptosis is one of the most studied (Rathmell and Thompson, 2002). In all vertebrates, the process of apoptosis is fundamental for proper development (Honarpour et al., 2001; Ke et al., 2018), tissue homeostatis (Eischen et al., 2001), and cancer prevention (Luke et al., 2003).

The BCL2 family is a group of proteins vital for regulating the intrinsic pathway of apoptosis (Czabotar et al., 2014; Singh et al., 2019), autophagy (Murakawa et al., 2015; Pedro et al., 2015), regulation of calcium signaling and homeostatis (Eckenrode et al., 2010; Eno et al., 2012), anti-inflammatory response (Badrichani et al., 1999), cell cycle control (Thomas et al., 2010), lipid metabolism (Escudero et al., 2018), and several DNA repair mechanisms (Hou et al., 2007; Wang et al., 2008; Kumar et al., 2010).

## The BCL2 Family

Human proteins belonging to this family can be sorted into two subgroups, according to whether they perform an anti-apoptotic or pro-apoptotic function. For example, the anti-apoptotic BCL2 proteins include the funding member Apoptosis regulator BCL2 (Tsujimoto et al., 1985), BCL2-like protein 1, BCL2L1 or BCLXL (Boise et al., 1993), Induced myeloid leukemia cell differentiation protein, MCL1 (Zhou et al., 1997), BCL2-like protein 2, BCL2L2 or BCLW (Gibson et al., 1996), BCL2-related protein A1, BCL2A1 or BFL1 (Lin et al., 1993), BCL2-like protein 10, BCL10 or BCLB (Lee et al., 2001). On the other hand, the pro-apoptotic family members comprise BCL2 associated X apoptotic regulator, BAX (Oltval et al., 1993), BCL2 homologous antagonist/killer 1, BAK1 (Chittenden et al., 1995), BCL2-related ovarian killer protein, BOK (Hsu et al., 1997), BCL2-like protein 15, BCL2L15 or BFK (Coultas et al., 2003), and BCL2-like protein 14, BCL2L14 (Guo et al., 2001). Another member of the BCL2 family, i.e., BCL2-like protein 13, BCL2L13 is involved both in mitochondrial fragmentation and mitophagy (Murakawa et al., 2015).

Despite their opposing roles in the context of apoptosis, most proteins of both groups share four homology domains, named BCL2 Homology domains (or BH) and numbered one to four (BH1, BH2, BH3, BH4) (Singh et al., 2019). Their overall three-dimensional (3D) structure includes two central hydrophobic α-helices (α5 and α6) surrounded by five amphipathic α-helices (α1- α4 and α7). The BH1, BH2, and BH3 motifs are arranged into a hydrophobic groove, while the BH4 motif lies at the N-terminus (Birkinshaw and Czabotar, 2017).

In addition, BCL2, BCLXL, MCL1, and BCLW share a C-terminal transmembrane domain which targets them to the mitochondrial outer membrane (MOM) (Warren et al., 2019). Other proteins seem to share a similar transmembrane domain for MOM targeting, such as the BH3-interacting domain death agonist, BID, and the proteins BCL2-like protein 11, BCL2L11 or BIM; BCL2-binding component 3, BBC3 or PUMA and Phorbol-12-myristate-13-acetate-induced protein 1, PMAIP1 or NOXA (Wilfling et al., 2012).

Furthermore, a recent study proposed that a region of BID is involved in the ability of the protein to induce MOM polarization (MOMP) independently from BID activation of BAK1 and BAX. According to this new mechanism, the region encompasses the helix α6 of BID, which is the structural equivalent of the helix α5 in BAK1 and BAX for their pore-forming activity (Flores-Romero et al., 2022). The structural details of this mechanism remain unclear and need to be further investigated.

BCL2, BCLXL, and MCL1 also feature an unstructured loop of approximately 60 residues connecting the helices α1 and α2, as well as separating the BH4 and BH3 motifs (Kelekar and Thompson, 1998). In BCL2, this loop can undergo phosphorylation at multiple sites (T56, S70, T74, S87) in response to different external stimuli (Breitschopf et al., 2000; Huang and Cidlowski, 2002; Bassik et al., 2004). The corresponding loop in BCLXL also harbors phosphorylatable sites (S49, S62) of functional relevance, whose state correlates with BCLXL’s activity in cell cycle progression (Wang et al., 2011) and sensitivity to microtubule-damaging drugs (Basu and Haldar, 2003; Upreti et al., 2008).

Two exceptions to the canonical structure of BCL2 proteins are BCL2L15 and BCL2L10. BCL2L15 possesses an overall architecture similar to the other folded BCL2 proteins but lacks the BH1 and BH4 domains (Coultas et al., 2003). BCL2L10 also deviates from the standard pattern, bearing a BH1 and a BH2 motif (Aouacheria et al., 2001; Zhang et al., 2001) but it does not include a BH3 motif (Rautureau et al., 2010).

BCL2 proteins are generally found in metazoan phyla (Aouacheria et al., 2013) even in ancient evolutionary species such as sponges (Wiens et al., 2001), and hydra (David et al., 2005), with the BCL2 family probably involved in a primitive immune response mechanism in cnidaria (Lasi et al., 2010). However, BCL2 proteins have also been found in numerous viruses (Cheng et al., 1997), which use molecular mimicry of BCL2 proteins as a survival strategy (Cuconati and White, 2002). While differences can be observed in the number of α-helices composing the structure of viral BCL2 proteins, all of them display an overall fold similar to that of human BCL2 proteins (Kvansakul and Hinds, 2013). An example of viral BCL2 comprising eight α-helices (as its human counterpart) is the apoptosis regulator BCL2 homolog found in the Kaposi sarcoma-associated virus, KSHV BCL2 (Cheng et al., 1997). Overexpression of KSHV BCL2 exerts an anti-apoptotic effect in cells infected with the Sindbis virus. It does not form homo- or heterodimers with BCL2, BCLXL, BAK, or BAX, suggesting an anti-death activity of KSHV BCL2 which is independent of these interactions (Cheng et al., 1997). However, a more recent report has shown that KSHV BCL2 binds the BH3 motif of BAK1 with nanomolar affinity and that of BAK with near-micromolar affinity (i.e., 980 nM). At the structural level, the BH3 peptides of BAK1 and BAX interact with the BH3-binding groove of KSHV BCL2 (Huang et al., 2002). The BCL2 homologs BHRF1 and BALF1 in the Epstein-Barr virus also harbor eight α-helices and exert an anti-apoptotic function upon various stimuli (Foghsgaard and Jäättelä, 1997; Kvansakul et al., 2010). DPV022, FPV039, and A179L are also anti-apoptotic BCL2 homologs containing eight helices, found in deerpox virus, fowlpox virus, and African swine fever virus, respectively (Burton et al., 2015; Anasir et al., 2017; Banjara et al., 2017). The murine γ-herpesvirus 68 (γHV68) also possesses a BCL2 homolog, HV68 BCL2, which blocks apoptosis in cultured cells under several pro-apoptotic stimuli (Loh et al., 2005). HV68 BCL2 contains seven α-helices and a groove responsible for the interaction with the BH3 motifs of BAK1 and BAX, which occurs in the low micromolar range (Loh et al., 2005). Other proteins from other viruses have been found with prosurvival activity and variation in the number of α-helices but depending on the binding to BAK1 and BAX (Kvansakul et al., 2007; Suraweera et al., 2020). Another BCL2-like protein, protein N1, has been found in vaccinia virus and can homodimerize and bind peptides containing the BH3 motifs of BID, BIM, and BAK1, whereas it does not interact with BAD (Aoyagi et al., 2007). Furthermore, the binding of N1 to the BH3 motif of BAX has been shown to inhibit apoptosis (Postigo et al., 2006). However, these interactions require further investigation in the context of the whole proteins since, for instance, when using full-length variants the capability of observing binding seems to be altered, while the N1-BAD interaction is observed (Cooray et al., 2007). Furthermore, Protein F1 from vaccinia virus is another anti-apoptotic protein showing a BCL2-like fold, plus a 60-residues-long N-terminal extension with a Caspase-9 inhibitory function and a role in inflammasome regulation (Kvansakul and Hinds, 2013). While F1 from vaccinia virus inhibits apoptosis in a BIM-dependent manner, F1 from variola virus exerts its anti-apoptotic function independently from BIM (Marshall et al., 2015). Finally, other viral proteins from herpesviruses or adenoviruses display anti-apoptotic activity and are functional BCL2 homologs (Chiou et al., 1994; Nava et al., 1997; Aouacheria et al., 2003).

## The BH3-Only Proteins and the Role of Their BH3 Motifs

Proteins not belonging to the BCL2 family can also have a BH3 motif. These proteins are defined as BH3-only and usually act as activators of apoptosis. They do so by binding the anti-apoptotic members of the BCL2 family, preventing them from sequestering the pro-apoptotic BCL2 proteins (Huang and Strasser, 2000). As a result, activated pro-apoptotic BCL2 proteins undergo dramatic conformational changes. These include the dissociation of helix α1 (Griffiths et al., 1999), which causes the exposure of the BH3 motif (Dewson et al., 2008). Furthermore, this also causes the release of the BCL2 core domain, which is formed by the helices α2 to α5, from the latch domain formed by the helices α6-ɑ8 (Czabotar et al., 2013; Brouwer et al., 2014). Hence, the core domains form symmetric homodimers where the helix α2 of one chain can engage the BH3 binding groove of another chain of a prosurvival BCL2-like protein. These BH3-in-groove dimers are the critical infrastructure to initiate the formation of pores in the mitochondrial outer membrane (Dewson et al., 2008; Bleicken et al., 2010; Czabotar et al., 2013; Aluvila et al., 2014; Brouwer et al., 2014; Subburaj et al., 2015). Pore formation initiates the process of MOMP (Bleicken et al., 2010). MOMP will then lead to the release of cytochrome c and other signaling factors and ultimately activate the caspase cascade and cell death (Singh et al., 2019).

An alternative mode of action describes BH3-only proteins as direct interactors of the pro-apoptotic BCL2 proteins. According to this model, BH3-only proteins are classified as either “activators” or “sensitizers” of apoptosis based on their ability to activate their pro-apoptotic counterparts directly. In this model, “sensitizers” can only sequester anti-apoptotic BCL2 proteins (Letai et al., 2002; Kuwana et al., 2005; Du et al., 2011; Czabotar et al., 2013). It has been suggested that “activators” bind to a rear pocket in BAX, distinct from the canonical BH_3_-binding groove, but the mechanistic details of this interaction remain unclear (Gavathiotis et al., 2008; Kim et al., 2009). However, other biochemical studies postulated that some BH3 motifs activate MOM-tethered BAK1 by interacting with its BH3-binding site (Dai et al., 2011). Furthermore, a survey demonstrated that the BH3 motifs of most BH3-only proteins could activate BAX and BAK1 when substituted with the BH3 motif of BID in BID/other-BH3-only chimeric proteins (Hockings et al., 2015). The entire direct-activation model has also been recently disputed (Huang et al., 2019), leaving the question of the exact mechanisms of action of BH3-only proteins open (Westphal et al., 2014). Furthermore, emerging evidence suggests that BH3-only proteins also act as sentinels of apoptosis, reporting the status of several cellular processes to BCL2 proteins (Moldoveanu et al., 2014).

Anti-apoptotic BCL2 proteins have also been shown to bind to BAK1 and BAX and retrotranslocate them from the mitochondria to the cytosol, preventing pore formation on the MOM (Edlich et al., 2011; Schellenberg et al., 2013; Todt et al., 2015). Mutational studies have suggested that this interaction is dependent on the BH3 motifs of BAX and BAK1 (Edlich et al., 2011).

The BH3 domain, therefore, is critical in mediating the balance shift between the activity of anti-apoptotic BCL2 proteins (which are sequestered after binding the BH3-only proteins) and the pro-apoptotic members of the family. Furthermore, it plays a crucial role in the dimerization of BAX during apoptosis. In detail, the interaction involves one BAX monomer interacting with the BH3 domain of the partner *via* its BH3-binding groove and vice versa (Dewson et al., 2012).

This review aims to clarify the key features of BH3 motifs and the structural determinants of currently known BH3-mediated interactions and their implication for structural studies and identification of new binding motifs.

BH3 motifs are found both inside and outside of the BCL2 protein family.

**FIGURE 1:**
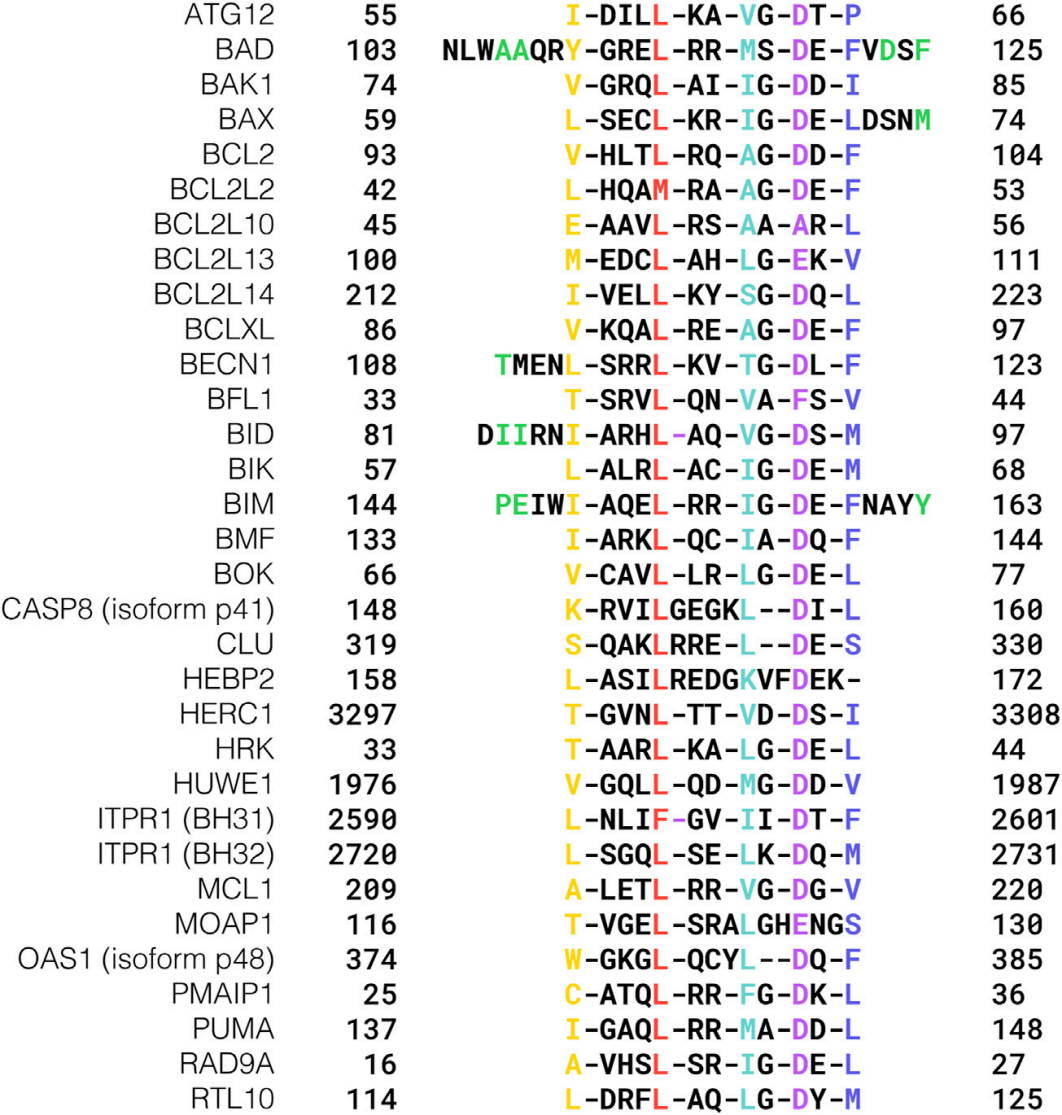
Core sequences of experimentally validated BH3 motifs. Conserved hydrophobic positions are highlighted in yellow, red, mint green, and blue, respectively, while the acidic hotspot is colored purple. BH3s whose flanking regions are examined in the text are reported in their extended version, and the residues discussed are colored green.

Initially, the BH3 motif was defined as a region with length varying between seven and fifteen residues, similar in sequence among the BCL2 proteins BCL2, BCLXL, BAK1, and BAX, plus a shorter, non-BCL2 protein called BIK (Kvansakul and Hinds, 2015). The BH3 motif has then been found in the BCL2 protein BID (Wang et al., 1996) and in several non-BCL2 proteins (Table 1; Figure 1), such as BIM (O’Connor et al., 1998), PUMA (Nakano and Vousden, 2001), NOXA (Oda et al., 2000), and BAD (Yang et al., 1995). For an extended list see Table 1. Furthermore, a BH3 motif was found in the E3 ubiquitin ligase HUWE1 with substrate recognition capabilities for MCL1 polyubiquitination (Zhong et al., 2005), showing that in at least one reported case the BH3 motif can undertake different tasks apart from the canonical death-ligand function.

**TABLE 1:** Experimentally validated BH3 motifs from human proteins. We included all members of the BCL2 family (both anti-apoptotic and pro-apoptotic) and BH_3_-containing interactors whose BH3s were reported as not dispensable for binding BCL2 proteins through deletion of the motif or mutations in the critical hotspots. The “PMIDs” column contains the PubMed IDs of the publications where BH3-mediated interactions involving the proteins of interest were described or where the functional role of such interactions was investigated (or both). The protein identifiers used throughout the text and in the figures are reported in parentheses in the first column, after the UniProt name and the isoform specification (if available). For each interaction mentioned in the column “Role of the BH3-mediated interactions involving the protein”, the two binding partners are separated by a slash, and the one whose BH3 motif mediates the interaction is written first.

UniProt name	UniProt	Gene name	BH3 start	BH3 end	BH3 sequence	PMID	Role of the BH3-mediated interactions involving the protein
2′-5′-oligoadenylate synthase (isoform p48) (OAS1)	P00973-3	*OAS1*	374	385	WGKGLQCYLDQF	11323417	Pro-apoptotic BH3-containing proteins/Anti-apoptotic BCL2 proteins balance: - OAS1/BCL2 [11323417] - OAS1/BCLXL [11323417]
Activator of apoptosis harakiri (HRK)	O00198	*HRK*	33	44	TAARLKALGDEL	9130713	Pro-apoptotic BH3-containing proteins/Anti-apoptotic BCL2 proteins balance - HRK/BCL2 [9130713] - HRK/BCLXL [9130713]
Apoptosis facilitator BCL2-like protein 14 (BCL2L14)	Q9BZR8	*BCL2L14*	212	223	IVELLKYSGDQL	11054413	Pro-apoptotic BH3-containing proteins/Anti-apoptotic BCL2 proteins balance - BCL2L14/BCLXL [11054413]
Apoptosis regulator BAX (BAX)	Q07812	*BAX*	59	70	LSECLKRIGDEL	11060313	Directly pro-apoptotic (BAX in-groove dimerization) [23374347]
						12242151	Directly pro-apoptotic (BH3-only binds BAX)
						16987815	- BID/BAX [16987815,18195012,23374347]
						18195012	- BIM/BAX [12242151,23374347]
						19380879	- MOAP1/BAX [11060313]
						21199865	- NOXA/BAX [23374347]
						23055042	Directly anti-apoptotic (anti-apoptotic BCL2 proteins sequester BAX)
						23374347	- BAX/BCLXL [21199865,27198225]
						23340338	- BAX/MCL1 [21199865]
						23374347	The BH3-dependence of the BOP/BAX interaction (which promotes apoptosis) is dubious [23055042]
						27198225	Contrasting evidence on a direct activation of BAX by PUMA [19380879,23340338]
Apoptosis regulator BCL2 (BCL2)	P10415	*BCL2*	93	104	VHLTLRQAGDDF	8918887	Pro-apoptotic BH3-containing proteins/Anti-apoptotic BCL2 proteins balance
						9130713	- BAD/BCL2 [12242151]
						10620799	- BIM/BCL2 [17115033]
						11323417	- BID/BCL2 [8918887]
						11546872	- BMF/BCL2 [11546872]
						12242151	- HRK/BCL2 [9130713]
						17115033	- OAS1/BCL2 [11323417]
						21199865	- RAD9A/BCL2 [10620799]
						22152474	Contrasting evidence on BID's ability to sequester BCL2 [8918887,18195012]
						23055042	Role of the (BH3-mediated) ATG12/B2 interaction in apoptosis remains to be investigated [22152474] The role of the (BH3-mediated) BOP/BCL2 interaction in apoptosis remains to be investigated [23055042]
BCL2 homologous antagonist/killer (BAK1)	Q16611	*BAK1*	74	85	VGRQLAIIGDDI	9020082	Directly pro-apoptotic (BAK1 homodimerizes and heterodimerizes with BAX) [15901672]
						12242151	Directly pro-apoptotic (the BH3-only proteins bind BAK1)
						15901672	- BIM/BAK1 [12242151]
						23055042	- BOP/A [23055042]
						25246614	- Caspase-8 isoform 41 [25246614]
						25408501	Indirectly anti-apoptotic (HERC1 ubiquitinates BAK1 causing its subsequent degradation) [25408501]
BCL2-binding component 3 (PUMA)	Q9BXH1	*BBC3*	75	86	IGAQLRRMADDL	11463392	Pro-apoptotic BH3-containing proteins/Anti-apoptotic BCL2 proteins balance
						19652530	- PUMA/BCLxL [19652530,23340338] which induces the release of pro-apoptotic p53 [23340338])
						19380879	Contrasting evidence on a direct activation of BAX by PUMA [19380879, 23340338]
						23340338	
BCL2-interacting killer (BIK)	Q13323	*BIK*	57	68	LALRLACIGDEM	9305912	The (BH3-mediated) interaction of BIK with BCL2 and BCLXL is insufficient to induce apoptosis [9305912]
BCL2-like protein 1 (isoform BCLXL) (BCLXL)	Q07817-1	*BCL2L1*	86	97	VKQALREAGDEF	9130713	Directly anti-apoptotic (BCLXL sequesters BAX) [21199865,27198225]
						9305851	Directly anti-apoptotic (TPT1 activates BCLXL) [26813996]
						9388232	Pro-apoptotic BH3-containing proteins/Anti-apoptotic BCL2 proteins balance
						10620799	- BAD/BCLXL [9305851,9388232]
						11323417	- BCLXS/BCLXL [11687955]
						11687955	- BID/BCLXL [18195012]
						17115033	- BIM/BCLXL [17115033]
						17446862	- HRK/BCLXL [9130713]
						18195012	- OAS1/BCL [11323417]
						19652530	- PUMA/BXL [19652530,23340338]
						21199865	- RAD9A/BCLXL [10620799]
						21639858	Regulation of Ca2+ signaling (ITPR1 binds to BCLXL) [26976600]
						23055042	Autophagy inhibition (BCLXL sequesters BECN1) [17446862]
						23340338	The (BH3-dependent) BECN1/BCLXL interaction does not affect BCLXL's anti-apoptotic function [30827509] The role of the (BH3-mediated) HEBP2/BCLXL interaction in apoptosis remains to be investigated [21639858] The role of the (BH3-mediated) BOP/BCLXL interaction in apoptosis remains to be investigated [23055042]
						25371206	
						26813996	
						26976600	
						27198225	
						30827509	
BCL2-like protein 1 (isoform BCLXS) (BCLXS)	Q07817-2	*BCL2L1*	86	97	VKQALREAGDEF	26146385	BH3 motif required for mitochondrial fragmentation, but BH3-mediated interactions remain to be investigated [26146385]
BCL2-like protein 2 (BCL2L2)	Q92843	*BCL2L2*	42	53	LHQAMRAAGDEF	18195012	Pro-apoptotic BH3-containing proteins/Anti-apoptotic BCL2 proteins balance
						23055042	- BID/BCL2L2 [18195012]
							The role of the (BH3-mediated) BOP/BCL2 interaction in apoptosis remains to be investigated [23055042]
BCL2-like protein 10 (BCL2L10)	Q9HD36	*BCL2L10*	45	56	EAAVLRSAAARL	23235460	The role of the (BH3-mediated) BIM/BCL2L10 interaction in apoptosis remains to be investigated [23235460]
BCL2-like protein 11 (BCL2L11)	O43521	*BCL2L11*	148	159	IAQELRRIGDEF	9430630	Directly pro-apoptotic (BIM binds the pro-apoptotic BCL2 proteins)
						12242151	- BIM/BAK1 [12242151]
						17115033	- BIM/BAX [12242151, 23374347]
						18195012	Pro-apoptotic BH3-containing proteins/Anti-apoptotic BCL2 proteins balance
						22040025	- BIM/BCL2 [17115033, 18195012]
						23235460	- BIM/BCLXL [17115033, 18195012]
						23374347	- BIM/MCL1 [17115033, 18195012]
						25907960	- BIM/BCL2L2 [18195012]—BIM/BFL1 [18195012] The role of the (BH3-mediated) BCL2/BCL2L10 interaction in apoptosis remains to be investigated [23235460]
BCL2-like protein 13 (BCL2L13)	Q9BXK5	*BCL2L13*	100	111	MEDCLAHLGEKV	26146385	BH3 motif required for mitochondrial fragmentation, but BH3-mediated interactions remain to be investigated [26146385]
BCL2-modifying factor (BMF)	Q96LC9	*BMF*	133	144	IARKLQCIADQF	11546872	Pro-apoptotic BH3-containing proteins/Anti-apoptotic BCL2 proteins balance—BMF/BCL2 [11546872]
BCL2-related ovarian killer protein (BOK)	Q9UMX3	*BOK*	66	77	VCAVLLRLGDEL	9804769	The role of possible BOK-BH3 mediated interactions in apoptosis remains to be investigated [9804769]
BCL2-related protein A1 (BFL1)	Q16548	*BCL2A1*	33	44	TSRVLQNVAFSV	23055042	Pro-apoptotic BH3-containing proteins/Anti-apoptotic BCL2 proteins balance—BID/BFL1 [18195012] The role of the (BH3-mediated) BOP/BFL1 interaction in apoptosis remains to be investigated [23055042]
BCL2-associated agonist of cell death (BAD)	Q92934	*BAD*	110	121	YGRELRRMSDEF	9305851	Pro-apoptotic BH3-containing proteins/Anti-apoptotic BCL2 proteins balance
						9372935	- BAD/BCLXL [9305851,9388232]
						9388232	- BAD/BCL2 [12242151]
						12242151	
BECN1	Q14457	*BECN1*	112	123	LSRRLKVTGDLF	17446862	Autophagy inhibition (BCLXL sequesters BECN1) [17446862]
						17659302	The (BH3-dependent) BECN1/BCLXL interaction does not affect BCLXL's anti-apoptotic function [30827509]
						17337444	
						19180116	
						30626284	
						30827509	
BH3-interacting domain death agonist (BID)	P55957	*BID*	86	97	IARHLAQVGDSM	8918887	Directly pro-apoptotic (BID binds BAX) [16987815,18195012,23374347]
						16987815	Pro-apoptotic BH3-containing proteins/Anti-apoptotic BCL2 proteins balance
						18195012	- BID/BCLXL [18195012]
						23374347	- BID/BCL2L [18195012]
						25408501	- BID/BLF1 [18195012]
						25907960	Contrasting evidence on BID's ability to sequester BCL2 [8918887,18195012]
Caspase-8 (isoform p41)	Q14790	*CASP8*	148	160	KRVILGEGKLDIL	25246614	Directly pro-apoptotic (Caspase-8 isoform p41 binds BAK) [25246614]
Cell cycle checkpoint control protein RAD9A (RAD9A)	Q99638	*RAD9A*	16	27	AVHSLSRIGDEL	10620799	Pro-apoptotic BH3-containing proteins/Anti-apoptotic BCL2 proteins balance
							- RAD9A/BCL2 [10620799]
							- RAD9A/BCLXL [10620799]
Clusterin (CLU)	P10909	*CLU*	319	330	SQAKLRRELDES	21527247	Pro-apoptotic BH3-containing proteins/Anti-apoptotic BCL2 proteins balance
						21567405	- Clusterin/BCLXL [21567405]
Cyclin-dependent kinase 4 inhibitor C (INK4C)	P42773	*CDKN2C*	156	145	GNAQMLSVVENR	32111816	Cell cycle regulation by the (BH3-mediated) INK4C/MCL1 interaction [32111816]
E3 ubiquitin-protein ligase HUWE1 (HUWE1)	Q7Z6Z7	*HUWE1*	1976	1987	VGQLLQDMGDDV	15989957	Indirectly pro-apoptotic (HUWE1 ubiquitinates MCL1 causing its subsequent degradation) [15989957]
Heme-binding protein 2 (HEBP2)	Q9Y5Z4	HEBP2	158	172	LASILREDGKVFDEK	21639858	The role of the (BH3-mediated) HEBP2/BCLXL interaction in apoptosis remains to be investigated [21639858]
Induced myeloid leukemia cell differentiation protein MCL1 (MCL1)	Q07820	*MCL1*	209	220	ALETLRRVGDGV	15694340	Pro-apoptotic BH3-containing proteins/Anti-apoptotic BCL2 proteins balance
						15989957	- NOXA/MCL1 [15694340]
						20562877	Indirectly pro-apoptotic (HUWE1 ubiquitinates MCL1 causing its subsequent degradation) [15989957]
						22152474	Cell cycle regulation by the (BH3-mediated) INK4C/MCL1 interaction [32111816]
						23055042	The role of the (BH3-mediated) BOP/MCL1 interaction in apoptosis remains to be investigated [23055042] The BH3-dependence of the ATG12/MCL1 interaction (which promotes apoptosis) is yet to be proven [22152474]
Inositol 1,4,5-trisphosphate receptor type 1 (ITPR1)	Q14643	*ITPR1*	2590 2720	2601 2731	LNLIFGVIIDTF LSGQLSELKDQM	26976600	Regulation of Ca2+ signaling (ITPR1 binds to BCLXL) [26976600]
Modulator of apoptosis 1 (MOAP1)	Q96BY2	*MOAP1*	116	130	TVGELSRALGHENGS	11060313	Directly pro-apoptotic (MOAP1 binds BAX) [11060313]
Phorbol-12-myristate-13-acetate-induced protein 1 (NOXA)	Q13794	*PMAIP1*	25	36	CATQLRRFGDKL	15694340	Directly pro-apoptotic (NOXA binds to BAX) [23374347]
						20392693	Pro-apoptotic BH3-containing proteins/Anti-apoptotic BCL2 proteins balance
						23374347	- NOXA sequesters MCL1 [15694340]
Probable E3 ubiquitin-protein ligase HERC1 (HERC1)	Q15751	*HERC1*	3297	3308	TGVNLTTVDDSI	25408501	Indirectly anti-apoptotic (HERC1 ubiquitinates BAK causing its subsequent degradation) [25408501]
Protein BOP (BOP)	Q7L3V2	*RTL10*	114	125	LDRFLAQLGDYM	23055042	Directly pro-apoptotic (BOP binds BAK) [23055042]
							The role of the (BH3-mediated) BOP/BCL2, BOP/BCLXL, BOP/MCL1, BOP/BCL2L2, and BOP/BFL1 interactions in apoptosis remains to be investigated [23055042]
							The BH3-dependence of the BOP/BAX interaction (which promotes apoptosis) is dubious [23055042]
Translationally-controlled tumor protein (TPT1)	P13693	*TPT1*	17	27	IYKIREIADGL	26813996	Directly anti-apoptotic (TPT1 activates BCLXL) [26813996]
Ubiquitin-like protein ATG12 (ATG12)	O94817	*ATG12*	55	66	IDILLKAVGDTP	22152474	The role of the (BH3-mediated) ATG12/BCL2 interaction in apoptosis remains to be investigated [22152474]
							The BH3-dependence of the ATG12/MCL1 interaction (which promotes apoptosis) is yet to be proven [22152474]

The Inositol 1,4,5-trisphosphate receptor type 1 protein ITPR1 also displays two functional BH3 motifs (Yang et al., 2016), which are reported as BH31 and BH32 in Figure 1. On the other hand, the BH3 motif found in the BCL2/adenovirus E1B 19 kDa protein-interacting protein 3-like BNIP3L/NIX does not bind any of the anti-apoptotic BCL2 proteins and does not activate BAX or BAK1 (Certo et al., 2006). Intriguingly, BNIP3L/NIX is involved in the binding with the BCL2 associated athanogene (BAG) family, which is a group of co-chaperones with different functionalities in quality control pathways, such as autophagy and proteasomal degradation (Pattingre and Turtoi, 2022).

Other putative BH3 motifs in the apolipoproteins or in the charged multivesicular body protein 5 and BCL2L15 have been deemed non-functional (Coultas et al., 2003; DeBartolo et al., 2014; Galindo-Moreno et al., 2014; Ultsch et al., 2021). The BH3 of the murine variant Bcl2l14 has also been classified as a non-functional motif (Giam et al., 2012), while the same motif is functional in the human variant BCL2L14 (Guo et al., 2001).

Other BH3s, such as the one found in the cell death regulator Aven, have been found to interact with BCL2 proteins *in vitro*, but their role in a cellular context remains to be elucidated (Liu et al., 2005; DeBartolo et al., 2014). Similar evidence can be found for other proteins, in which a proapoptotic function has been found but the detail of the interactions remains to be established (Broustas et al., 2004; Strohecker et al., 2008).

The BH_3_ motif of vesicle transport protein SEC20 or BNIP1 also mediates its apoptotic activity (Yasuda and Chinnadurai, 2000). However, BNIP1 variants harboring deletions of the motif still interact with BCLXL, suggesting that more than one region may be responsible for the binding of BNIP1 to BCL2 proteins (Yasuda and Chinnadurai, 2000). A similar scenario was observed in BNIP3, where mutants lacking its BH3 domain were still able to bind BCL2 (Ray et al., 2000).

Furthermore, a class of proteins containing functional BH3 motifs is constituted by the products of splicing variants of the *BCL2L1* and *MCL1* genes. They produce the anti-apoptotic proteins BCLXL and MCL1, and their BH3-only, pro-apoptotic counterparts, i.e., BCLXS, MCL1S, and MCL1ES (Bae et al., 2000; Plötz et al., 2012; Warren et al., 2019). The *BCL2* gene also produces two isoforms of BCL2, with BCL2α constituting the canonical one and BCL2β lacking a long C-terminal region with respect to BCL2α (residues 196–239). Instead, the *BAX* gene produces six additional isoforms: BAXβ, BAXγ, BAXδ, BAXε, BAXσ, and BAXΔ2. BAXβ and BAXγ were identified in the same study (Oltval et al., 1993). BAXβ lacks the residues 159–192, which are substituted by 60 additional residues, while BAXγ lacks the residues 42–192 and has a different sequence in the region comprised between positions 12 and 41 with respect to the main *BAX* isoform due to a deletion which causes a reading frameshift. Both BAXβ and BAXγ contain a BH_3_ domain, whose role in their activity has not been investigated yet. BAXδ contains the BH1, BH2, and transmembrane domains, but it does feature any BH_3_, since the region corresponding to positions 30–78 of the main BAX isoform is missing (Apte et al., 1995). BAXε, despite lacking the BH_2_ and transmembrane domains, shows pro-apoptotic activities and it is able to homodimerize and heterodimerize with BCL2, BCLXL, and the canonical BAX isoform (Shi et al., 1999). BAXσ lacks the region 159–171 of the main BAX isoform (between the BH2 and the transmembrane domains) and harbors a BH_3_ motif (Schmitt et al., 2000), but its role in the pro-apoptotic function of BAXσ remains unclear. On the other hand, BAXΔ2 was identified in several cancer cell lines not expressing the main BAX isoform. The BAX gene isolated from these cell lines contains a single deletion in exon 3, which causes a reading frame shift and subsequent premature termination of the transcript, resulting in the expression of the BAXΔ2 isoform. BAXΔ2 can homodimerize and heterodimerize with BCL2 and induces cell death without directly targeting mitochondria, possibly through a mechanism involving the downstream effector caspase-8 (Haferkamp et al., 2012). BAXΔ2 also contains a BH3 motif, but its function in BAXΔ2-induced apoptosis remains yet to be elucidated.

## BH3 Motifs Have Features of Short Linear Motifs

BH3s have been recently proposed as an instance belonging to the class of Short Linear Motifs (SLiMs) (Aouacheria et al., 2015). Indeed, BH3s do not exhibit significant sequence conservation. They usually share two residues (a leucine and an aspartate) separated by a variable number of non-conserved residues (Aouacheria et al., 2015). The borders of BH3 motifs are also poorly defined, some of them encompassing 20-residues-long stretches (DeBartolo et al., 2014).

Evolutionarily, recent evidence indicates that the BH3 motifs currently observed may be the result of processes of divergent, random, and convergent evolution, together with transfer events, which may explain their heterogeneity (Aouacheria et al., 2015). In addition, the motifs in BH3-only proteins are disordered when the proteins are in their unbound states (Hinds et al., 2007) and seem to fold in helical conformation upon binding, even if this is not templated by the partner protein and encoded in the BH3 sequence (Crabtree et al., 2018). In light of the above observations, BH3 motifs, especially in BH3-only proteins, seem to share many commonalities with SLiMs (Davey et al., 2012; van Roey et al., 2014).

## Isolated BH3 Motifs are Mostly Disordered in Solution but Fold Into α-Helices When Binding BCL2 Proteins

We collected all the known structures in the Protein Data Bank for BH3-containing proteins in complex with BCL2-like proteins (Supplementary Table S1). The details of commonalities and different nuances in the binding modes are reported in this Section.

BH3 domains are mostly disordered in solution but interact with their BCL2 family partners as α-helices (Hinds et al., 2007), showing different propensities for adopting this class of secondary structure (Petros et al., 2000, 2004; Dahal et al., 2018). Furthermore, a computational study has shown that the BH3-only protein PUMA favors molten-globule-like conformations in solution (Chebaro et al., 2014). At the same time, significant energy barriers exist between the entirely folded state of the α-helix and the partially folded states (Chebaro et al., 2014). Many new and more tailored force fields for disordered proteins have been developed since this study (Best, 2017). The tendency to achieve a molten-globule-like conformation should be reassessed with these new physical models due to the tendency of old force fields to cause over-compaction of IDPs. As mentioned above, it has been suggested that binding pathways of BH3-only proteins to their BCL2 partners are encoded within their intrinsically disordered regions and not templated by the partner (Crabtree et al., 2018).

Further biophysical and simulation studies could shed light on the propensity of the free state of the protein for the formation of helical structures and the differences in free energies between the different states. The community has made substantial progress in the development of the force field for simulations of disordered proteins that can more accurately capture the ensembles observed in solution with experimental methods (Best, 2017; Huang and MacKerell, 2018; Bhattacharya and Lin, 2019), along with enhanced sampling approaches that can better cover the heterogeneous ensembles attained by disordered regions in solutions (Bussi and Laio, 2020).

Different studies have also demonstrated that the helicity of BH3 peptides positively correlates with their binding affinity for their BCL2 family counterparts (Petros et al., 2000; Hinds et al., 2007; Lama and Sankararamakrishnan, 2011). For this reason, the helical structure of bound BH3 peptides has prompted the development of synthetic peptides that can mimic the naturally occurring BH3 helices, as detailed below. Several strategies have been employed to produce stable BH3-mimicking helices, ranging from different kinds of residue cross-linking (Okamoto et al., 2013), to the insertion of α- and β-amino acids (Horne et al., 2008; Boersma et al., 2012; Peterson-Kaufman et al., 2015). Furthermore, specific mutations have been found to enhance the binding between BCL2 proteins and BH3 motifs, possibly stabilizing the α-helical conformation (Petros et al., 2000). Methods such as molecular simulations or biophysical approaches such as NMR and circular dichroism could help to confirm or disprove this hypothesis at the structural level.

**FIGURE 2:**
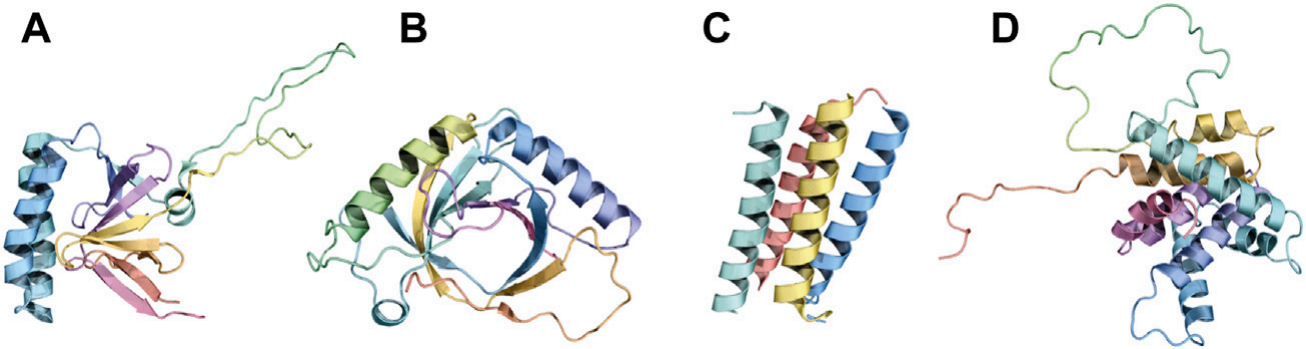
Folded BH3-only proteins and bundling of BH3 motifs of BIM. Panels **(A)**, **(B)**, and **(D)** depict the fold of the BH3-only proteins TPT1 (PDB ID: 2HR9), HEBP2 (PDB ID: 3R8J), and BID (PDB ID: 2BID), respectively. Panel **(C)** illustrates the tetrameric association between four BH3 peptides of BIM (PDB ID: 6X8O).

A recent work demonstrated that 26- and 27-residues long BH_3_ peptides from BIM can oligomerize in solution. In the oligomers, BIM molecules can assume a packed conformation of two sets of parallel α-helices, the two sets being antiparallel to each other (set 1: peptides A, D, set 2: peptides B, C, Figure 2C). π-stacking interactions seem to be especially relevant for maintaining the overall architecture of this peculiar multi-BH3 structure, with W147 from each peptide π-stacked against Y162 of its neighbor, and Y162 in peptide C involved in another π-stacking interaction with Y163 of peptide B (Assafa et al., 2021).

BID, HEBP2, and TPT1 represent three exceptions to the unfolded-in-solution paradigm. BID is a globular protein that displays a fold similar to that of multi-domain BCL2 proteins (Figure 2D), with the BH3 domain packed against the core with its hydrophobic face buried (Chou et al., 1999). On the other hand, HEBP2 displays an entirely different fold, resembling an open distorted β-barrel composed of eight anti-parallel β-strands. Two α-helices (Figure 2B) connect the second to the third β-strand and the sixth to the seventh β-strand. HEBP2 requires extensive conformational changes to be able to bind BCLXL (Ambrosi et al., 2011). TPT1 is also structured. The peculiarity of TPT1 in the context of BCL2 proteins resides in its BH3 region. Indeed, TPT1’s BH3 motif is composed of β-strands, while it assumes a helical conformation (Figure 2A) upon binding with BCLXL (Thebault et al., 2016).

**FIGURE 3:**
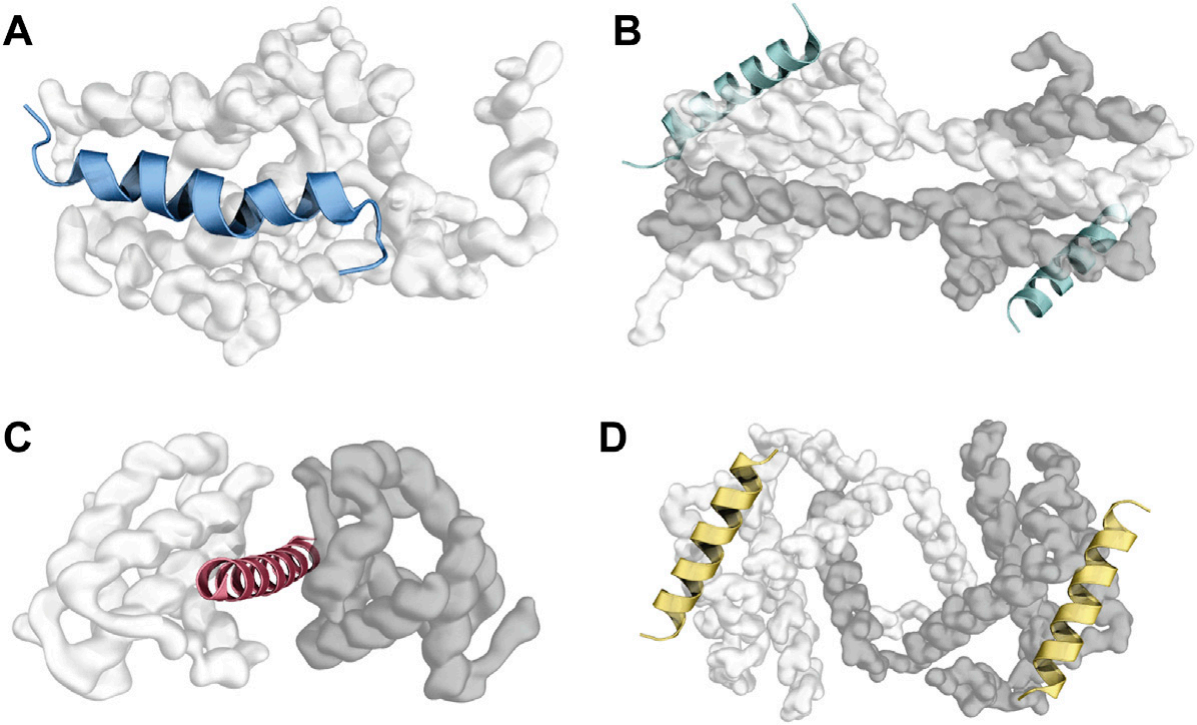
Different topologies were observed in crystals of human BCL2 protein/BH3 motif complexes. **(A)** represents the canonical topology, with one molecule of the BCL2 protein bound to a single BH3 peptide (PDB ID: 2M04). Panel **(B)** and **(D)** illustrate the “core/latch” and “helix ɑ1” dimers, respectively, where two intertwined molecules of the BCL2 protein engage two BH_3_ motifs (one each) with their BH3-binding grooves (PDB IDs: 5VWW and 6DCN). Panel **(C)** depicts the “sandwiched” conformation, where one BH3 motif is squeezed between two monomers of the BCL2 protein (PDB ID: 3PL7).

It is worth mentioning that not all solved structures of BCL2 protein/BH_3_ motif complexes present the same canonical topology (Figure 3A, Supplementary Table S1). For example, in a fraction of solved crystal structures of BCL2 proteins in a complex with BH3 motifs, the BCL2 proteins present in one asymmetric unit of the crystal may adopt a peculiar topology known as “helix- α1-swapped dimer” (Ambrosi et al., 2011; Thebault et al., 2016). Here, the two monomers of the BCL2 protein exchange their N-terminal helix α1 (Figure 3D). This conformation is likely a crystallization artifact in human proteins, and it seems to not influence the binding mode of BH3s in the canonical binding groove. However, a modified version of this swapped dimeric configuration, adopted by the viral proteins F1 and DPV022, is likely to be functional since the dimer is the molecular species of F1 and DPV022 observed in solution (Kvansakul et al., 2008; Burton et al., 2015).

Another conformation that BCL2 proteins can adopt in crystals is the so-called “core/latch dimer” (Figure 3B), where helices α5, α6, α7, and α8 in one monomer extend and interact with the corresponding helices in the second monomer (Follis et al., 2013; Brouwer et al., 2014, 2017). This dimeric structure of BCL2 proteins does not alter their interaction with BH_3_ motifs either (O’Neill et al., 2006; Brouwer et al., 2017). Czabotar and coworkers also identified a third unusual topology involving one BAX-BH3 peptide “sandwiched” between two BCLXL molecules (Figure 3C). Here, the Bax-BH3 motif is inserted in the BH3-binding groove of one of the BCLXL monomers while contacting a previously unknown interface on the other monomer. However, the authors suggested this interface to be a crystallization artifact (Czabotar et al., 2011). Finally, interactions between BCL2 proteins and BH3 motifs are heavily influenced by the cellular environment, with different interaction patterns observed in solution and in the membranes (Das et al., 2017).

## FOUR HYDROPHOBIC AND ONE ACIDIC RESIDUES IN BH3 MOTIFS ARE ESSENTIAL FOR THE INTERACTION WITH BCL2 PROTEINS (CANONICAL BINDING MODE)

**FIGURE 4:**
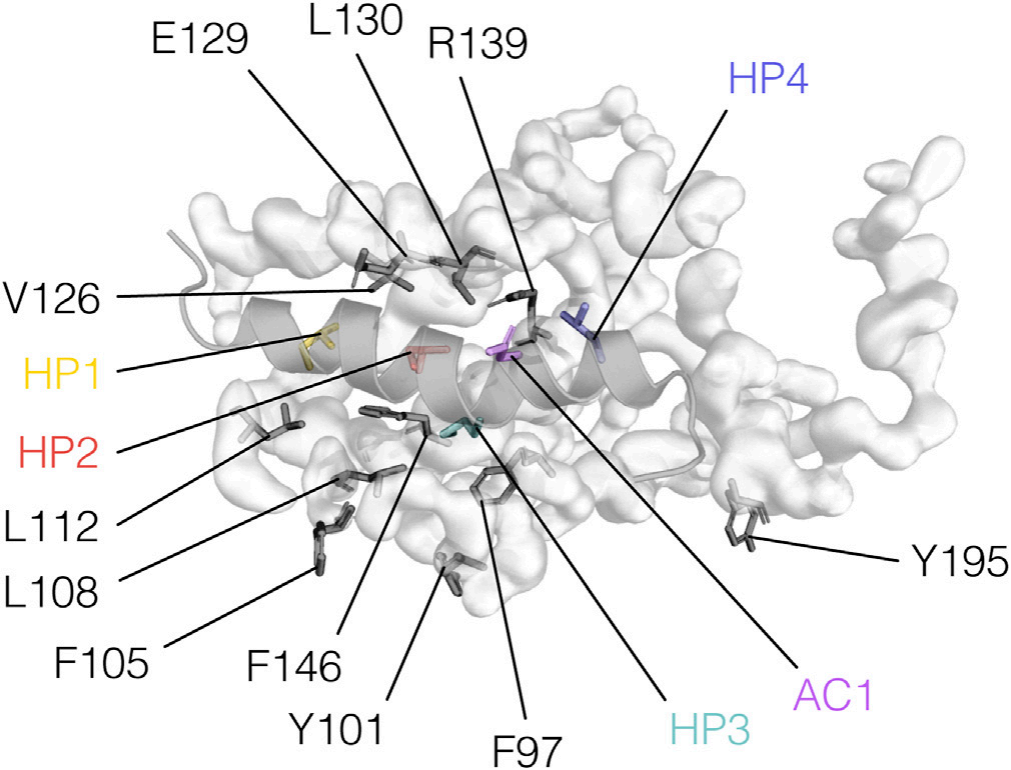
BH3 motif hotspots and residues relevant to their interaction with BCL2 proteins. BH3 hotspots and BCL2 residues are illustrated on the BCLXL/PUMA-BH3 complex (PDB ID: 2M04) and color-coded as in Figure 1.

The mode of interaction between BCL2 proteins and BH3 motifs is conserved among the family members, consisting of the hydrophobic face of an amphipathic helix formed by a BH3 motif binding into a hydrophobic groove formed by the BH1, BH2, and BH3 motifs of the BCL2 protein (Petros et al., 2000; Liu et al., 2003; Czabotar et al., 2007). A patch of four hydrophobic residues (Figure 4), hereafter termed HP1, HP2, HP3, and HP4 and aligned on a “stripe” along the surface-exposed face of the BH3 helix, is essential to such interaction. Another crucial position, located between HP3 and HP4, is usually occupied by acidic residues and will therefore be referred to as AC1 (Figure 4). We recently showed that this position could tolerate substitutions to asparagine or glutamine which could retain the needed electrostatic interaction for the binding of a BH3 region with BCL2-like proteins (Kønig et al., 2019).

Alanine substitutions at the conserved sites in a BCLXL/BAK1-BH_3_ complex (L78A, I81A, I85A, D83A, positions HP2, HP3, HP4, AC1, respectively) destabilize the binding (Sattler et al., 1997). According to the same study, alanine substitutions of HP1 (V74) are also disruptive. However, a leucine-to-phenylalanine mutant of BIM-BH3 at HP2 (L152 F) can still tightly binds BCLXL, suggesting that some modifications may be tolerated even at conserved positions (Lee et al., 2009). These hotspot residues may also be relevant in modulating the specificity of BH_3_ domains for their BCL2 family interaction partners, as discussed later. For example, it has been postulated that position HP2 may be crucial for distinguishing pro-survival from pro-apoptotic BCL2 family members (Kelekar and Thompson, 1998).

In BCL2 proteins, residues important for interacting with BH3 motifs are mostly located in the BH1, BH2, and BH3 regions (Figure 3). For instance, arginine residues in the BH1 motifs of BCLXL, MCL1, BCL2L2, BFL1, BAX or BAK are important for interactions with different partners (Sattler et al., 1997; Denisov et al., 2006; Feng et al., 2007; Oberstein et al., 2007; Herman et al., 2008; Stewart et al., 2010; Rajan et al., 2015; Thebault et al., 2016).

The residue E129 of BCLXL is also involved in electrostatic interactions with residues mostly located within the core BH3 sequence (Sattler et al., 1997; Ambrosi et al., 2011; Rajan et al., 2015; Thebault et al., 2016). However, Oberstein and coworkers reported E129 as contacting an arginine C-terminal to the core motif of BECN1 as well (Oberstein et al., 2007). The fine details of the network of hydrophobic interactions are best understood in complexes of BH_3_ motifs with BCLXL, given the abundance of structural data. Here, interactions with the four hydrophobic hotspots are usually mediated by F97, Y101, F105, L108, V126, L130, F146, and Y195 (Sattler et al., 1997; Petros et al., 2000; Ambrosi et al., 2011; Thebault et al., 2016). On the other hand, L112 seems to form contacts, especially with the BH_3_ motifs of BECN1 and HEBP2 (Feng et al., 2007; Ambrosi et al., 2011; Lee et al., 2019).

## Residues Outside the Five Canonical Hotspots can Contribute to the Interaction Between BH3 Motifs and Multidomain BCL2 Proteins

Additional positions in BH3 motifs can be relevant to their interaction with BCL2 proteins.

For example, the position just preceding AC1 (AC1-1) is occupied by glycine in most BH3s (Figure 1) and mutating it to aspartate in the BH3 motif of BECN1 leads to a dramatic decrease of affinity for BCLXL (Oberstein et al., 2007).

Moreover, a hydrophobic residue N-terminal to HP1 in BID (I82/I83, termed HP0) has been found to govern the ability of the protein to activate BAX (Czabotar et al., 2013; Robin et al., 2015). Furthermore, alanine mutations in the same positions in IM, occupied by P144 and E145, result in a three-fold loss of affinity for BAX, corroborating the hypothesis that HP0 plays a significant role in several BH3 motifs (Robin et al., 2015). Furthermore, A106 and A107 in BAD in position HP0 contact BCLXL in the structure solved by Petros and coworkers (Petros et al., 2000). However, mutating those two residues to glycine does not impact BAD’s interaction with BCLXL (Petros et al., 2000). In contrast, position HP2+1 in BIM (R153) establishes additional interactions with BAX and accounts for a five-fold increase in the binding affinity of BIM with respect to BID (Robin et al., 2015).

The BH_3_ motif of BAX also displays a nonstandard position affecting its binding to other BCL2 proteins. Indeed, M74 in HP4+4 was found to interact with the BH3-binding groove of pro-survival BCL2 proteins, mutating this position to alanine, arginine, lysine, aspartate, and glutamate dramatically impairs the interaction of BAX-BH3 with BCLXL, BCL2, and BCL2L2 (Czabotar et al., 2011). Furthermore, BAX mutants at M74 reduce cell viability and enhance susceptibility to ABT-737, an antagonist of BCL2, BCLXL, and BCL2L2, highlighting the functional significance of alterations at this position (Czabotar et al., 2011). Moreover, Y163 in the BH_3_ motif of BIM, corresponding to BAX M74, is found interacting with BFL1 in a crystal structure of the complex (Herman et al., 2008). Position HP4+4 of BAD (F125) has also been found contacting BCLXL in the structure solved by Petros and coworkers (Petros et al., 2000). In the same work, the authors also postulated that a mutation to arginine of D123, at position HP4+2, enhances the binding affinity of BAD for BCLXL by stabilizing the helical conformation of the peptide.

Post-translational modifications within the BH3 motif can also modulate its ability to bind BCL2 proteins and to act in the apoptotic process. For instance, a mutation of the conserved acidic position in the BH3 motif of BAD (D119G) promotes the pro-apoptotic activity of the protein by preventing BAD phosphorylation at multiple sites, some of which are in the vicinity of the motif (Adachi et al., 2003). Furthermore, a recent study investigated the structural mechanisms underlying the increase, albeit weak, in binding affinity observed upon phosphorylation of the BH3 of BECN1 (at T108) bound to BCL2 and BCLXL (Lee et al., 2019). However, given the differences observed when the interaction was studied in solution and in membrane surrogates, more work is needed to confirm the structural determinants of this phosphorylation event.

Phosphorylation can also allosterically regulate BCL2-like proteins, as attested by the example of phosphorylations in the disordered long loop of BCLXL that reduce its antiapoptotic activity. In this mechanism, allostery acts in two directions, i.e., inducing a direct displacement of p53 from BCLXL and long-range displacements of the bound BH3-only partners, as well as in the opposite direction directly displacing BH3-only protein and allosterically remodeling the distal site, causing a displacement of p53 (Follis et al., 2018).

## BH3 Motifs can be Promiscuous or Show Binding Preferences to a Subset of BCL2 Proteins

While some pro-apoptotic BCL2 proteins, like BIM, BID, and PUMA bind to all anti-apoptotic BCL2 family members (Chen et al., 2005; Certo et al., 2006; Ku et al., 2011), others only interact with specific subsets of them.

For instance, BAD interacts strongly with BCL2, BCLXL, and BCL2L2, weakly with BFL1, and cannot recognize MCL1 (Chen et al., 2005; Certo et al., 2006). On the other hand, NOXA binds only to MCL1 and BFL1 (Chen et al., 2005). Finally, BMF interacts with BCL2, BCLXL, BCL2L, and MCL1, but not with BFL1. In contrast, HRK seems to be specific to BCLXL (Certo et al., 2006) and could bind BCL2A1 (Kønig et al., 2019). Among the pore-forming pro-apoptotic effectors, BAX binds to all anti-apoptotic family members, while BAK1 only interacts with BCLXL and MCL1 (Willis et al., 2005).

Furthermore, a wide array of affinities is observed for BMF peptides when binding different BCL2 family members, with interactions between BMF peptides and MCL1 and BFL1 being three to four orders of magnitude weaker than interactions of the same peptides with BCL2, BCLXL, and BCL2L2 (Chen et al., 2005).

The BH_3_ peptides from BAD also display preferences for BCL2, BCLXL, and BCL2L2 over MCL1 and BFL1 (Chen et al., 2005; Kuwana et al., 2005; Certo et al., 2006; Boersma et al., 2008; Lee et al., 2008). Regarding the biochemical details of such preferences, an investigation of the binding kinetics of several BH_3_ peptides to MCL1 showed that the variations observed in affinity were primarily due to differences in the dissociation constants more than in the association rates (Dahal et al., 2018).

Several studies have also addressed the effects of substitutions in BH_3_ domains on the interaction with different BCL2 family members, with a few focusing on the differences in the interaction of BH3 motifs with BCLXL versus MCL1 (Boersma et al., 2008; Lee et al., 2008).

These investigations have shown, for instance, that the mutated BH3 peptides from BIM, including those with two or three alanine mutations at conserved hydrophobic hotspots, maintain a high binding affinity for MCL1. At the same time, that for BCLXL drops dramatically (Lee et al., 2008).

Moreover, a comprehensive study by Dutta and coworkers based on a high-throughput, yeast surface display-based approach identified mutations that can selectively impact the binding of BIM with BCLXL and MCL1 (Dutta et al., 2010). For instance, isoleucine in position HP2 was found to be highly specific for MCL1. Other critical insights include the constrain of the HP3 position primarily to wild-type isoleucine for MCL1 specific peptides and peptides that are bound to both proteins. In contrast, sequences bound solely to BCLXL displayed a wider array of preferences. A difference in structural packing of the HP3 position, tightly inserted into the MCL1 pocket but close to the more flexible region between helices α2 and α3 in BCLXL, may be the underlying cause of the observed permissiveness. A substitution of BAK1 I181 with phenylalanine has also been found to enhance the affinity to BCLXL (Chin and Schepartz, 2001), further highlighting the relevance of HP3 for binding specificity.

In position HP4, sequences specific for BCLXL present large aromatic residues while MCL1 can also accommodate other substitutions (Dutta et al., 2010). A saturation mutagenesis study corroborates this finding (Lee et al., 2007). Moreover, mutating F159 (HP4) in the BH3 motif of BIM to valine increased the affinity of the peptide for MCL1 and conferred a binding preference for MCL1 over BCLXL. On the other hand, sequences featuring phenylalanine or tyrosine in HP1 interact specifically with BCLXL (Dutta et al., 2010).

Regarding position HP1, the BH3 motif of BAD features a tyrosine and is highly specific for BCLXL, and mutational studies have confirmed that this residue affects binding specificity (Day et al., 2005). Apart from the canonical hotspots, mutations to asparagine, aspartate, or glutamate of position HP2+1 are present in MCL1-specific sequences and absent from the BCLXL-specific ones, hinting at a novel putative determinant of binding specificity (Dutta et al., 2010).

## Some BH3 Motifs Display Non-Canonical Binding Modes

The plasticity of BH3-mediated interactions is further revealed by the discovery of BH_3_ motifs interacting with their BCL2 family counterpart through non-canonical binding modes. This plasticity is likely to be due to the high heterogeneity and pliability of the BH3-binding groove in BCL2 proteins, as shown for BCLXL (Follis et al., 2013; Liu et al., 2017).

An example is a BH3 motif found in the cell cycle regulatory protein INK4C which binds MCL1 in a reversed orientation with respect to that commonly observed for BH3 motifs. This interaction is of particular interest from a functional standpoint, since it provides a novel mechanism for MCL1-mediated modulation of cell cycle progression (Whitaker and Placzek, 2020).

**FIGURE 5:**
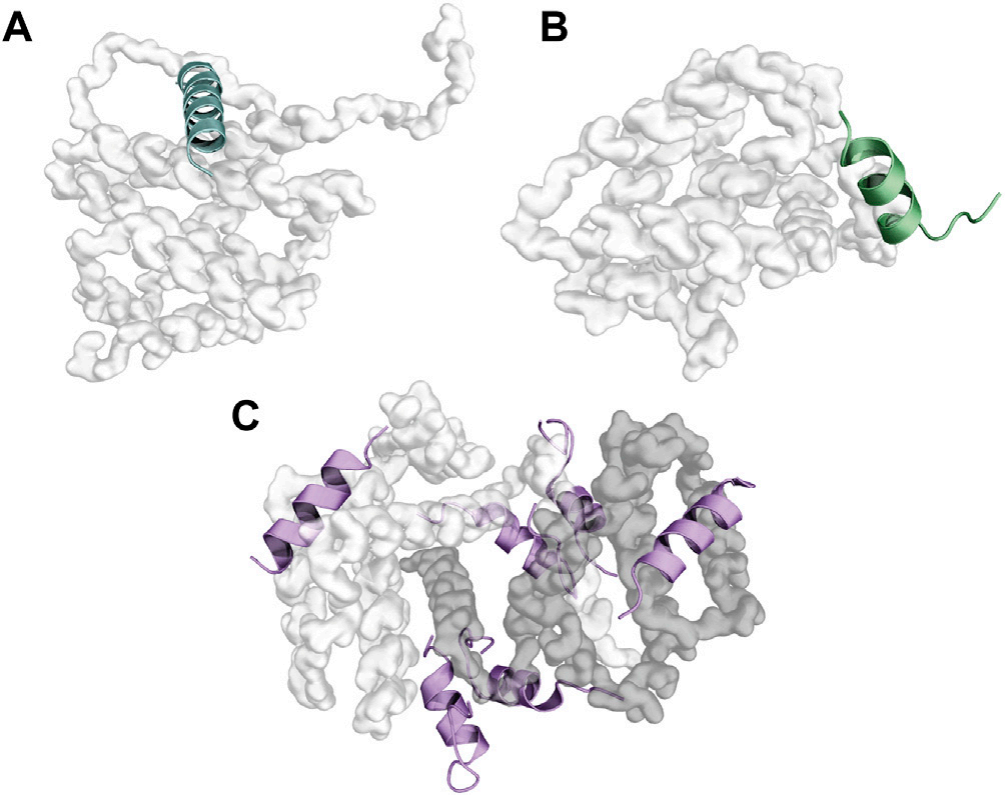
Examples of non-canonical binding modes involving either BH3 motifs or the BH3 binding groove. Panel **(A)** displays a helix-stabilized version of IM bound to a new interaction site on BAX (PDB ID: 2K7W), while panel **(B)** illustrates the binding of the non-BH3 interactor p73-TAD on BCLXL (PDB ID: 6IJQ). Finally, panel **(C)** shows the binding of the BH3 of TPT1 to BCLXL (PDB ID: 4ZD9). The BH3 peptides of TPT1 contacting BCLXL outside of the canonical binding groove are considered crystallization artifacts.

Another non-standard interaction mode is embodied by the BH3 motif of TPT1 bound to BCLXL. Here, the HP1 position is missing, while HP2 (I20), HP3 (I23), HP4 (L27), and AC1 (D25) bind into the canonical binding groove (Figure 5C). Furthermore, an alanine mutation of R21 at position HP1+1 abrogating the binding of the BH3 of TPT1 to BCLXL indicates that additional residues are critical for the interaction (Thebault et al., 2016). TPT1 is also the only protein known so far harboring a BH3-like motif that activates BCLXL instead of inhibiting it. Interestingly, replacing the region N-terminal to the HP2 position of the BH3 of TPT1 with the corresponding one of BAX completely abrogates the anti-apoptotic function of TPT1, suggesting that the non-standard binding mode may be responsible for the unusual behavior of TPT1 on apoptosis (Thebault et al., 2016).

BH_3_ motifs can also bind novel interaction sites on BCL2 proteins. For example, a helix-stabilized BIM-BH3 peptide binds into a previously unknown hydrophobic pocket on BAX, formed by helix α1 and α6 (Figure 5A). Noticeably, this interaction is sufficient to trigger apoptosis in retrovirally reconstituted *BAX* −/− *BAK* −/− mouse embryonic fibroblasts (Gavathiotis et al., 2008), suggesting a possible therapeutic potential for compounds targeting this alternate site to modulate apoptosis.

On the other hand, residues in the canonical BH3-binding groove on BCL2 proteins can also mediate interactions with non-BH3 partners, as has been shown (Yoon et al., 2018) for the TAD domain of p73 (Tumor protein p73) (Figure 5B). The interaction site of DJ1 (Parkinson disease protein 7) on BCLXL also partly overlaps with the BH3-binding groove (Lee et al., 2018).

Furthermore, the BH3 motif of MCL1 has been found to bind VLCAD (Very long-chain specific acyl-CoA dehydrogenase, mitochondrial) following a helix-in-groove pattern analogous to that observed for canonical BH3 binding to BCL2 proteins. The discovery of this interaction also highlighted a new role for MCL1 in the metabolism of fatty acids (Escudero et al., 2018). Finally, the BH3-binding site of BAX has also been found to accommodate its C-terminal hydrophobic α-helix, therefore allowing BAX to remain soluble in the cytoplasm (Suzuki et al., 2000).

The differences in the binding modes for the proteins mentioned in this Section are remarkable and many other proteins are yet to discover or clarify for their structural determinants of binding to BCL2 proteins. Moreover, the BH3-binding pocket of some members of the BCL2 family is highly dynamic and conformationally heterogeneous, as attested by BCLXL. This conformational heterogeneity, including also ordered/disordered transitions, could suggest that the pocket can recruit more interactors also beyond the BH3 class. We are still far from a complete understanding of their regulation and if they will act in the same way as known BH_3_ motifs on the BCL2 family members, or, alternatively, modulate other functions of this class of proteins. Examples such as the ones described in the previous sections on non-canonical BH_3_ motifs and the importance of positions outside the core regions point to the latter. In this context, we speculate that the interactors called ‘non-conventional’ BH_3_s could deviate substantially from the features of a BH3 to the point that we will need to consider different classifications in the future.

## THE BINDING OF BH3 MOTIFS CAUSES STRUCTURAL CHANGES IN BCL2 PROTEINS

**FIGURE 6:**
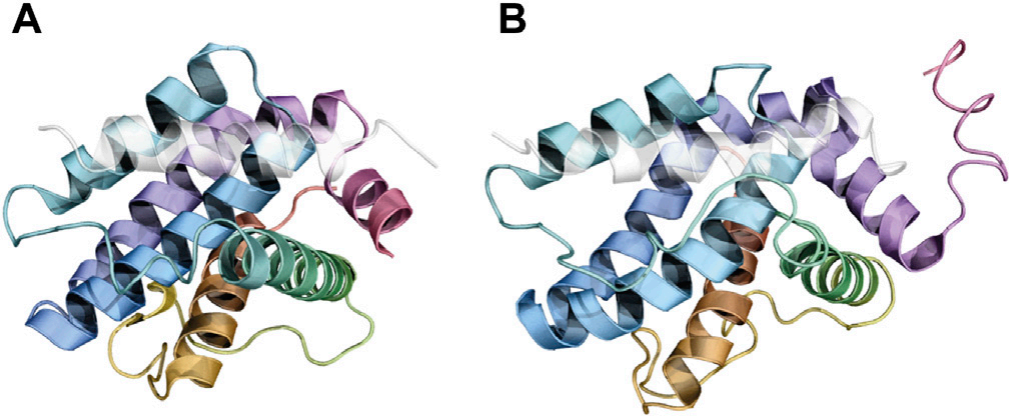
Conformational changes in helix ɑ3 of BCLXL induced upon BH3 binding. BCLXL is displayed in rainbow colors in both panels, while the BH3 peptide is colored light gray and semitransparent. The helix ɑ3 is shown in green. In panel **(A)**, the complex between BCLXL and the BH3 motif of BECN1 shows a folded ɑ-helix (PDB ID: 2PON), while a partial unfolding is observed in panel **(B)**, in the complex between the BCLXL and the BH_3_ of PUMA (PDB ID: 2M04).

Together with folding-upon-binding phenomena postulated for BH3 motifs when interacting with their BCL2 family counterparts, several observations suggest that structural perturbations occur also in the BH3-binding groove of BCL2 proteins because of this interaction. For example, solution structures of the dimer formed by BCLXL and PUMA have shown a partial unfolding of the helix α3 of BCLXL (Figure 6B), which in turn was postulated to trigger an allosteric communication to the p53-binding site of BCLXL, promoting the subsequent release of p53 (Cellular tumor antigen p53) and the initiation of apoptosis (Follis et al., 2013; Sora et al., 2021).

Further evidence of the intrinsic dynamics of helix α3 of BCLXL has been found in crystal structures of BCLXL bound to other BH3-containing proteins, such as BECN1 (Oberstein et al., 2007). In this case, the B-factors associated with α3 were generally higher than those observed for the other helices. Heterogeneity in the conformation of helix α3 was also observed in a complex between BCLXL and synthetic analogs of the BH3 motif of BIM (Boersma et al., 2012). The helix presents as highly disordered (despite visible electron densities) in a complex between BCL2L2 and its BH3 motif as well (Lee et al., 2014).

Helix α3 appears to be more ordered in complexes involving MCL1 suggesting different intrinsic dynamic properties of the two helices among BCL2 family members that may translate into functional differences. However, MCL1 also undergoes conformational changes upon binding BIM, resulting in the widening of the BH3-binding groove and the reorientation of the C-terminal portion of helix α4, which moves away from the BH3 peptide of BIM (Fire et al., 2010). The structural plasticity of MCL1 could help explain the accommodation of point mutants of BH3 motifs at the conserved hydrophobic hotspots. For instance, mutation of I148 of BIM to alanine causes a shift of L235 in MCL1 to fill the cavity created by the small residue (Fire et al., 2010), while an L152A/F159A double mutant results in a similar reorganization of M231 (Lee et al., 2008).

BAX also changes upon binding with BH3 motifs. In detail, several studies have highlighted the formation of a cavity between helices α2, α5, and α8 upon interaction with BH_3_ motifs. This cavity has not been observed in structures of either monomeric BAX or of complexes of other BCL2 proteins with BH3 motifs, and Czabotar and coworkers postulate it can destabilize BAX and promote extrusion of its BH3 domain, the first step towards oligomerization (Czabotar et al., 2013).

Finally, BH_3_-only proteins also cause allosteric changes in other BH_3_-only proteins. For instance, recent evidence pointed toward an allosteric mechanism governing the action of BAD on BID when they are both bound to multi-BCLXL complexes associated with membranes (Bogner et al., 2020). This discovery led to the formulation of a new model for BAX activation in membranes, where BID, sequestered by BCLXL multimers under physiological conditions, is activated during apoptosis thanks to BAD binding to the unoccupied BH_3_-binding groove of one of the BCLXL monomers of the complex. This activated form of BID could therefore activate the membrane-bound BAX.

## Design of BH3 Mimetics

A detailed structural understanding of the binding between BH3 motifs and their partners of interaction is fundamental not only from a basic research perspective but also in light of its applicative potential. The interactions between BCL2 proteins are therapeutically relevant and disruption of their complexes through competitive binding of small mimetics is the mechanism at the base of different pharmaceutical drugs, of which some are already in clinical trials (Delbridge and Strasser, 2015; Arulananda et al., 2020; Cerella et al., 2020). he rationale behind the design of drugs that act as BH_3_ mimetics is to provide small molecules that can directly activate apoptosis in tumor cells. The mimetics should bind and inhibit specific prosurvial BCL2 proteins, resembling the proapoptotic effect of BH3-containing proteins. An example is venetoclax, which has been approved by the US Food and Drug Administration and other authorities for the treatment of different leukemias. Other mimetics have been tested in preclinical cancer models but, in many cases, studies are still needed to ensure the proper balance between efficacy, specificity, and tolerability. Another important aspect that should not be neglected is the development of resistance to the BH3 mimetic drugs and investing efforts in the design of strategies to overcome it. A relevant and comprehensive review on this topic has been recently published by Diepstraten et al. (Diepstraten et al., 2022).

The emergence of other interactors for prosurvival BCL2 proteins, which do not obey the classical rules of a BH3-BCL2 interaction, could also provide different directions for drug design, depending on the understanding of the mechanism of action of these new classes of interactors.

## Open Research Questions

BH_3_ motifs represent a challenge for both experimental investigations and computational studies. Indeed, their sequence degeneration has already raised the question of whether a consensus motif can be inferred from the known motif instances since alignments of experimentally validated BH3s yield patterns with little to no predictive value in discovering new hits. The weak sequence conservation effectively hinders computational screenings, since too strict a definition would miss many functionally relevant entries while being too permissive would yield an unacceptably high number of false positives. Furthermore, the significant role played by flanking regions in modulating binding affinity or determining specificity makes the borders of the motif itself poorly defined. An additional layer of complexity is determined by post-translational modifications, whose presence should not be neglected when characterizing a new putative BH3 motif. From a structural standpoint, future research should incorporate the investigation of the dynamics of BH3 motifs both in solution and when bound to their BCL2 partners. For instance, it could be intriguing to further explore the intrinsic dynamics of portions of the binding groove, the formation of cavities, and the propagation of allosteric signals through an ensemble perspective. In this regard, molecular simulations and related computational techniques guided by available structural information could provide invaluable insights. Furthermore, the evaluation of BH3-mediated interactions in membranes, when possible, would provide a more accurate picture of how the central events of apoptosis unfold, and how they may intertwine with other cellular processes. In conclusion, both established, and more recent evidence reviewed here delineate multiple directions that novel studies could pursue, with the only certainty that the long-lasting research field revolving around BCL2 proteins and BH_3_-containing interactors is here to stay.
